# Magnetic Photocatalyst BiVO_4_/Mn-Zn ferrite/Reduced Graphene Oxide: Synthesis Strategy and Its Highly Photocatalytic Activity

**DOI:** 10.3390/nano8060380

**Published:** 2018-05-29

**Authors:** Taiping Xie, Hui Li, Chenglun Liu, Jun Yang, Tiancun Xiao, Longjun Xu

**Affiliations:** 1 State Key Laboratory of Coal Mine Disaster Dynamics and Control, Chongqing University, Chongqing 400044, China; deartaiping@163.com; 2 Chongqing Key Laboratory of Extraordinary Bond Engineering and Advanced Materials Technology (EBEAM), Yangtze Normal University, Chongqing 408100, China; 3 College of Chemistry and Chemical Engineering, Chongqing University, Chongqing 401331, China; lihui@163.com; 4 Chongqing Key Laboratory of Environmental Materials & Remediation Technologies Chongqing University of Arts and Sciences, Yongchuan 402160, China; yangjun@163.com; 5 Inorganic Chemistry Laboratory, University of Oxford, Oxford OX1 3QR, UK; xiaotiancun@chem.ox.ac.uk

**Keywords:** BiVO_4_, RGO, Mn–Zn ferrite, magnetic photocatalyst, magnetic performance, photocatalytic mechanism

## Abstract

Magnetic photocatalyst BiVO_4_/Mn-Zn ferrite (Mn_1−*x*_Zn_*x*_Fe_2_O_4_)/reduced graphene oxide (RGO) was synthesized by a simple calcination and reduction method. The magnetic photocatalyst held high visible light-absorption ability with low band gap energy and wide absorption wavelength range. Electrochemical impedance spectroscopies illustrated good electrical conductivity which indicated low charge-transfer resistance due to incorporation of Mn_1−*x*_Zn_*x*_Fe_2_O_4_ and RGO. The test of photocatalytic activity showed that the degradation ratio of rhodamine B (RhB) reached 96.0% under visible light irradiation after only 1.5 h reaction. The photocatalytic mechanism for the prepared photocatalyst was explained in detail. Here, the incorporation of RGO enhanced the specific surface area compared with BiVO4/Mn_1−*x*_Zn_*x*_Fe_2_O_4_.The larger specific surface area provided more active surface sites, more free space to improve the mobility of photo-induced electrons, and further facilitated the effective migration of charge carriers, leading to the remarkable improvement of photocatalytic performance. Meanwhile, RGO was the effective acceptor as well as transporter of photo-generated electron hole pairs. •O_2_^−^ was the most active species in the photocatalytic reaction. BiVO_4_/Mn_1−*x*_Zn_*x*_Fe_2_O_4_/RGO had quite a wide application in organic contaminants removal or environmental pollution control.

## 1. Introduction

In the recent decade, composite semiconductor materials are considered extraordinarily attractive in the field of solar energy and pollution control engineering. Many kinds of photocatalytic composite materials with superior optical properties and high photo-induced activity have been synthesized and studied [1,2]. However, the utilization efficiency of visible light for some photocatalysts is very low, owing to their large intrinsic band gap energy, which impels scientists to explore new photocatalytic compounds with high visible light-driven photocatalytic activity. Bismuth-based composites with *n*-type junctions exhibited excellent photocatalytic activity and high stability [3]. Among them, monoclinic crystal BiVO_4_, due to its relatively lower band gap energy has been of much interest in the photocatalysis field. Nevertheless, single component BiVO_4_ has poor absorption ability for visible light, leading to low quantum efficiency.

Meanwhile, the difficulty in separation and recovery for bismuth-contained photocatalysts greatly restricts their industrial application. Therefore, magnetic composite photocatalysts are vitally important in photocatalysis materials science, due to their simple recovery via an external magnet after reaction. Magnetic compounds, such as Fe_3_O_4_ and ZnFe_2_O_4_, have been extensively studied, due to their interesting properties, including photoactivity and stability. There are synthesis strategies and property studies for magnetic composite catalysts [4,5,6,7,8]. However, the recovery rate and the photocatalytic activity of these composites do not meet the need of industrial applications yet. Comparing magnetization and stability, Mn_1−*x*_Zn_*x*_Fe_2_O_4_ is superior to Fe_3_O_4_ and ZnFe_2_O_4_.

Reduced graphene oxide (RGO) possessing several good properties (e.g., electrical conductivity, optical transparency and carrier mobility) has been paid considerable attention [9,10,11]. Single layer graphene sheet is composed of sp^2^-hybridized carbon atoms in the two-dimension honeycomb lattice, which donates high mobility for electron carriers. The distinctive structure of RGO determines that its band gap energy is zero [12,13]. There are reports on the preparation method for RGO-composed catalysts and their activity [14,15,16]. It is reasonable to mingle RGO with BiVO_4_, which is aimed at enhancement of the migration rate of photo-produced electrons and holes of BiVO_4_. Previous investigation showed that BiVO_4_–RGO composite possessed photocatalytic performance and redox ability [17]. Unexpectedly, a larger visible light photocatalytic activity could not be observed in BiVO_4_–RGO system under visible light irradiation [18,19].

Here, fabrication of BiVO_4_/Mn_1−*x*_Zn_*x*_Fe_2_O_4_/RGO was a continuation of our research about the syntheses and application of BiVO_4_/Mn_1−*x*_Zn_*x*_Fe_2_O_4_ [20]. The RhB degradation reaction using BiVO_4_/Mn_1−*x*_Zn_*x*_Fe_2_O_4_ as photocatalyst was slow (take 3 h). The incorporation of RGO could boost the photocatalytic reaction kinetics. Here, the photocatalytic activity and mechanism are deeply investigated with RhB degradation and the radical capturing experiments using BiVO_4_/Mn_1−*x*_Zn_*x*_Fe_2_O_4_/RGO as photocatalyst.

## 2. Experimental Procedures

### 2.1. Preparation of BiVO_4_/Mn_1−x_Zn_x_Fe_2_O_4_/RGO

BiVO_4_/Mn_1−*x*_Zn_*x*_Fe_2_O_4_ was prepared according to our previous report [20]. Graphene oxide (GO) was fabricated with improving Hummars method [21].

GO (36.0 mg) and 1.2 g BiVO_4_/Mn_1−*x*_Zn_*x*_Fe_2_O_4_ were dispersed in deionized water with ultrasonication and stirring for 2 h. GO was reduced into RGO with NH_3_ H_2_O–N_2_H_4_ H_2_O solution (1.0 mL–3.0 mL), then filtered and washed four times with deionized water and ethanol before placing at 80 °C for 2 h. BiVO_4_/Mn_1−*x*_Zn_*x*_Fe_2_O_4_/RGO was obtained after drying at 60 °C for 24 h.

### 2.2. Materials Characterization

The phase and structure of samples were determined by X-ray Diffractometer (Shimadzu, XRD-6000, Kyoto, Japan), Fourier transform infrared spectroscopy (FTIR, Perkin-Elmersystem 2000, Akron, OH, USA), and INVIA Raman microprobe (Renishaw Instruments, Wotton-under-Edge, UK). The light absorption, magnetization, and surface performances of samples were examined by ultraviolet–visible diffuse reflectance spectrophotometer (UV–vis DRS, TU1901, Beijing, China), vibrating sample magnetometer (VSM 7410, LakeShore, Carson, CA, USA), Brunauer–Emmett–Teller (BET, ASAP-2020, Micromeritics, Norcross, GA, USA). The electrochemical workstation (PGSTAT30) was employed to measure electrochemical impedance spectroscopy (EIS) of the as-prepared samples. The test parameters of EIS were the following, K_3_[Fe(CN)_6_]/K_4_[Fe(CN)_6_] (1:1)—KCl electrolyte solution was employed. The work electrode content contained the as-produced catalyst, acetylene black, and polytetrafluoroethylene (mass ratio, 85.0:10:5), the counter electrode was platinum foils, and the reference electrode was the saturated calomel electrode (SCE), setting AC voltage amplitude of 5 mV and a frequency range of 1 × 10^5^–1 × 10^−2^ Hz.

### 2.3. Photocatalytic Activity, Stability, and Corresponding Mechanism

The photocatalytic activity of BiVO_4_/Mn_1−*x*_Zn_*x*_Fe_2_O_4_/RGO was investigated by the rhodamine B (RhB) degradation under visible light irradiation [22]. Ninety milligrams of composite photocatalyst (named fresh photocatalyst) was put into 5.0 mg/L RhB solution (100.0 mL). The solution was placed for 0.5 h with stirring in the dark to reach the adsorption–desorption equilibrium. A 500 W Xe lamp was used as the visible light source, equipped with ultraviolet (UV) light cut-off filter (λ ≥ 400 nm). At given irradiation time intervals, a series of the reaction solution was sampled and the absorption spectrum was measured.

The stability for the photocatalyst were assessed by cycling tests. After each cycle, the photocatalyst was separated and recovered by means of an external magnet. The recovered catalyst was respectively washed with ethanol and deionized water, then dried at the end of each cycle.

The photocatalytic mechanism of BiVO_4_/Mn_1−*x*_Zn_*x*_Fe_2_O_4_/RGO was explored by holes-radical trapping experiments with *p*-benzoquinone (BZQ,) (•O_2_^−^ radical scavenger), Na_2_-EDTA (hole scavenger), and *tert*-butanol (*t*-BuOH) (•OH radical scavenger) in photocatalytic reaction.

## 3. Results and Discussion

### 3.1. Optimal Synthesis Condition

The Mn_1−*x*_Zn_*x*_Fe_2_O_4_, prepared in advance, had a strong magnetization. In order to completely form BiVO_4_ precursor and reduce the impurity, Mn_1−*x*_Zn_*x*_Fe_2_O_4_ was put into the precursor instead of Bi(NO_3_)_3_ solution, in other words, BiVO_4_ precursor was already formed before magnetic substance was added. Mn_1−*x*_Zn_*x*_Fe_2_O_4_/BiVO_4_ was assembled via calcination at only 450 °C. This temperature was lower than that of Mn_1−*x*_Zn_*x*_Fe_2_O_4_ formation (1200 °C), as well as BiVO_4_ formation (500 °C). Therefore, the calcination approach was indeed low-cost and economical.

GO was dispersed in BiVO_4_/Mn_1−*x*_Zn_*x*_Fe_2_O_4_ with deionized water under room temperature, RGO was produced by NH_3_ H_2_O + N_2_H_4_ H_2_O reduction of GO without heating. This in situ synthesis method was simple and with low-energy consumption.

### 3.2. Structure and Phase Identification

**Figure 1:**
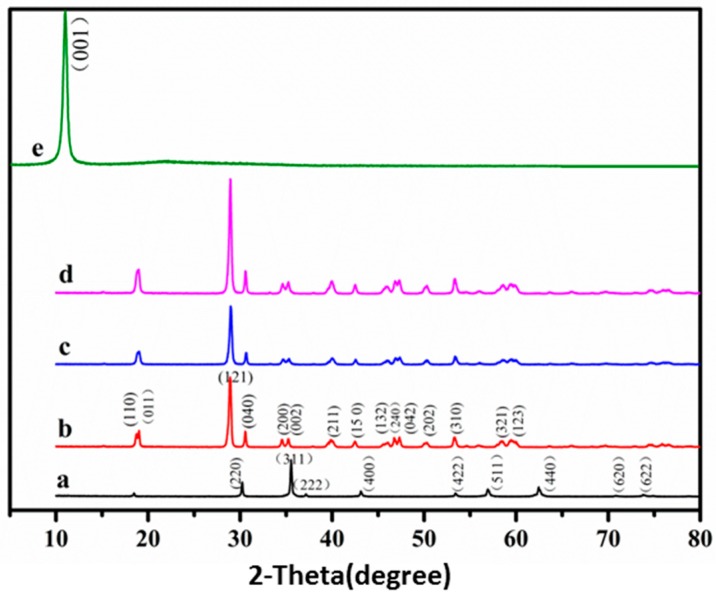
XRD patterns of (**a**) Mn_1−*x*_Zn_*x*_Fe_2_O_4_; (**b**) BiVO_4_; (**c**) BiVO_4_/Mn_1−*x*_Zn_*x*_Fe_2_O_4_; (**d**) BiVO_4_/Mn_1−*x*_Zn_*x*_Fe_2_O_4_/RGO (reduced graphene oxide); (**e**) GO (graphene oxide).

The XRD spectra of the obtained samples were shown in Figure 1. The characteristic spectra (Figure 1b–d) of monoclinic crystal BiVO_4_ was well indexed with the standard card (JCPDS card No: 14-0688) [17], corresponding to the diffraction phases of (110), (011), (121), (040), (200), (002), (211), (150), (132), and (042). The diffraction pattern in Figure 1a of Mn_1−*x*_Zn_*x*_Fe_2_O_4_ was fully matched with the standard card (JCPDS card No: 74-2400), agreeing with the result of the literature report [20]. The diffraction peaks of Mn_1−*x*_Zn_*x*_Fe_2_O_4_ patterns were hardly observed in Figure 1c,d. Not only was the amount (15.0%) of the magnetic matrix low, but also, the diffraction patterns location of Mn_1−*x*_Zn_*x*_Fe_2_O_4_ overlapped with the domain diffraction patterns of BiVO_4_. The diffraction peak of GO (Figure 1e) was observed at 10.8° (crystal plane (001)) [23]. However, the peak (Figure 1d) disappeared after GO was mostly reduced to RGO under the reduction of NH_3_ H_2_O and N_2_H_4_ H_2_O [24]. Moreover, the amount (3%, *w*/*w*) of RGO was not enough to be detected in X-ray diffraction. In short, it was deduced that the prepared samples totally exhibited good crystallinity.

**Figure 2:**
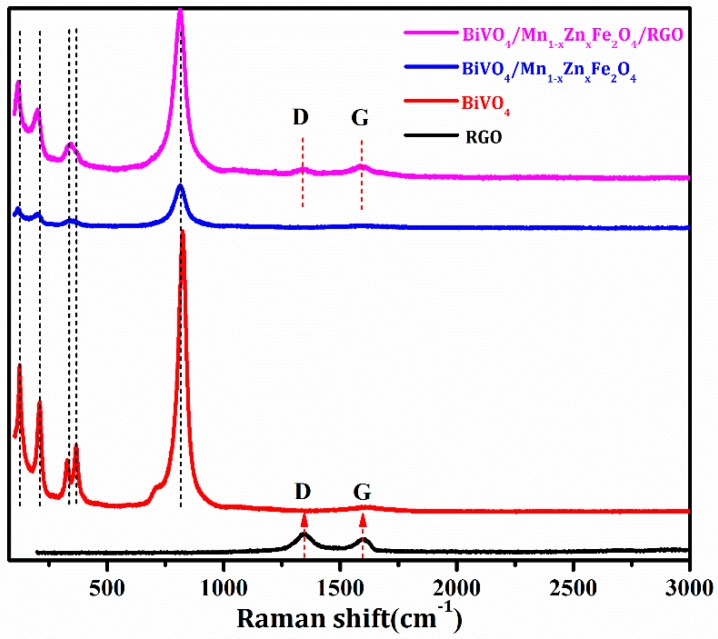
Raman spectra of RGO, BiVO_4_, Mn_1−*x*_Zn_*x*_Fe_2_O_4_/BiVO_4_, and Mn_1−*x*_Zn_*x*_Fe_2_O_4_/BiVO_4_/RGO.

The peak–intensity ratio (I_D_/I_G_) of D band (~1364.0 cm^−1^, originating from disorder-activated Raman mode) and G band (~1598.0 cm^−1^, corresponding to sp^2^ hybridized carbon) in RGO was usually used to assess the reduction extent. Figure 2 showed the Raman spectra of the above-obtained samples. It was seen that G-band of RGO was shifted from 1598.0 cm^−1^ to 1589.0 cm^−1^, while the D-band shorted from 1364.0 cm^−1^ to 1352 cm^−1^ after the thermal reduction finished. The I_D_/I_G_ ratio of GO was 1.10, and that of BiVO_4_/Mn_1−*x*_Zn_*x*_Fe_2_O_4_/RGO decreased to 0.84. The relative low I_D_/I_G_ ratio of RGO implied high reduction efficiency in BiVO_4_/Mn_1−*x*_Zn_*x*_Fe_2_O_4_/RGO [17]. Typical Raman bands of BiVO_4_ were located at 120.0, 210.0, 324.0, 366.0, and 826.0 cm^–1^ in Figure 2. The two bands at 324.0 cm^−1^ and 366.0 cm^−1^ changed into one wide band in BiVO_4_/Mn_1−*x*_Zn_*x*_Fe_2_O_4_, as well as in BiVO_4_/Mn_1−*x*_Zn_*x*_Fe_2_O_4_/RGO. The result was also consistent with the results of the previous report [25].

**Figure 3:**
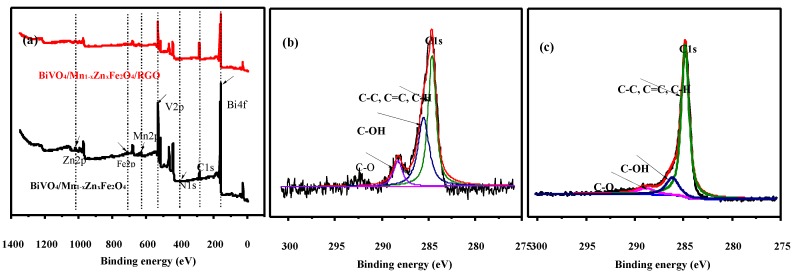
XPS survey spectra of (**a**) full range scan of BiVO_4_/Mn_1−*x*_Zn_*x*_Fe_2_O_4_ and BiVO_4_/Mn_1−*x*_Zn_*x*_Fe_2_O_4_/RGO; (**b**) C1s peaks in BiVO_4_/Mn_1−*x*_Zn_*x*_Fe_2_O_4_ (**c**) C1s peaks in BiVO_4_/Mn_1−*x*_Zn_*x*_Fe_2_O_4_/RGO.

To investigate the valence state and the surface property of BiVO_4_/Mn_1−*x*_Zn_*x*_Fe_2_O_4_/RGO, XPS spectrum characterization was employed. As displayed in Figure 3a, the spectrum intensity of C 1s in BiVO_4_/Mn_1−*x*_Zn_*x*_Fe_2_O_4_/RGO was larger than that in BiVO_4_/Mn_1−*x*_Zn_*x*_Fe_2_O_4_, namely, the introduction of RGO brought the intensity increase of C 1s. The spectrum intensity of oxygen-containing functional groups in Figure 3b was larger than that in Figure 3c, meaning the decrease of GO and the increase of RGO in BiVO_4_/Mn_1−*x*_Zn_*x*_Fe_2_O_4_/RGO sample. This feature confirmed the efficient reduction of GO and the valence states for various elements in BiVO_4_/Mn_1−*x*_Zn_*x*_Fe_2_O_4_/RGO.

**Figure 4:**
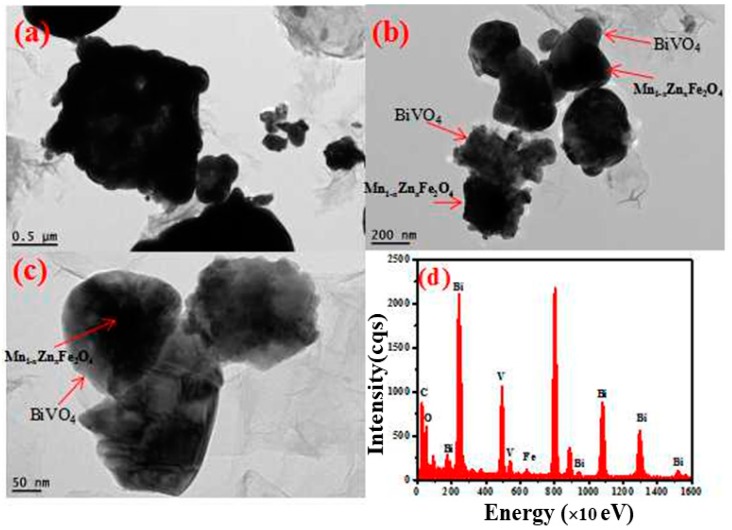
TEM images of Mn_1−*x*_Zn_*x*_Fe_2_O_4_/BiVO_4_/RGO with different resolution (**a**–**c**) and (**d**) EDS of Mn_1−*x*_Zn_*x*_Fe_2_O_4_/BiVO_4_/RGO.

Figure 4 was the transmission electron microscopy (TEM) images of the as-synthesized BiVO_4_/Mn_1−*x*_Zn_*x*_Fe_2_O_4_/RGO. In detail, there were the black core of Mn_1−*x*_Zn_*x*_Fe_2_O_4_ and the gray shell of BiVO_4_, and RGO sheets had good interfacial contact with BiVO_4_/Mn_1−*x*_Zn_*x*_Fe_2_O_4_ spherical particle. In other words, there was an overlap between BiVO_4_/Mn_1−*x*_Zn_*x*_Fe_2_O_4_ and RGO. At the same time, energy dispersive spectroscopy (EDS) of the composite revealed the presence of Fe, Bi, V, O, and C elements in Mn_1−*x*_Zn_*x*_Fe_2_O_4_, BiVO_4_, and RGO, which was in good agreement with XPS investigation.

**Figure 5:**
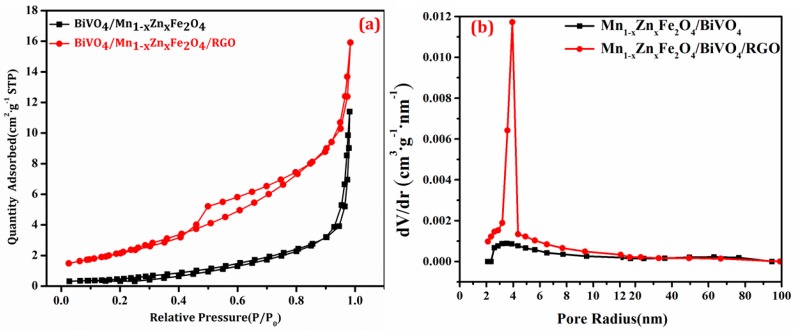
The adsorption–desorption isotherms of compounds (**a**), and the pore size distribution curves of compounds (**b**).

**Figure 6:**
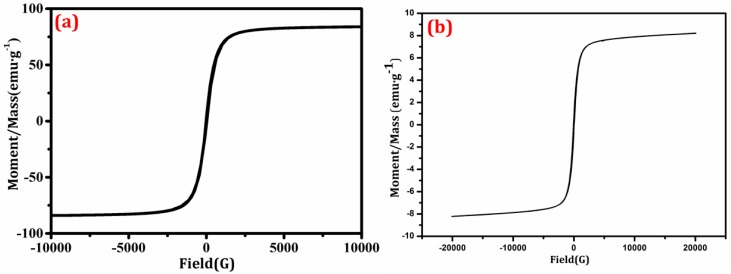
Hysteresis loops of products (**a**) Mn_1−*x*_Zn_*x*_Fe_2_O_4_; (**b**) BiVO_4_/Mn_1−*x*_Zn_*x*_Fe_2_O_4_/RGO.

Specific surface area of the as-obtained compounds was determined with the adsorption instrument, and the result was shown in Figure 5. The adsorption–desorption isotherms in Figure 5 were the typical isotherm III, agreeing with the reference report [26]. The discrete curve of BiVO_4_/Mn_1−*x*_Zn_*x*_Fe_2_O_4_/RGO was in p/p_0_ range of 0.45–0.55, and the pore diameter distribution was mainly 2–10 nm, and the most probable distribution was located in 4 nm. It was deduced that the introduction of RGO caused the mesopore increase and the macropore decrease. Thus, there was the uniform surface structure in the ternary composite. Calculating with the data in Figure 6, the specific surface area of BiVO_4_/Mn_1−*x*_Zn_*x*_Fe_2_O_4_/RGO was 8.84 m^2^/g, and that of BiVO_4_/Mn_1−*x*_Zn_*x*_Fe_2_O_4_ was only 2.22 m^2^/g. The incorporation of RGO enhanced the specific surface area compared with BiVO4/Mn_1−*x*_Zn_*x*_Fe_2_O_4_. The larger specific surface area provided more active surface sites, more free space to improve the mobility of photo-induced electrons, and further facilitated the effective migration of charge carriers, leading to the remarkable improvement of photocatalytic performance [27]. The surface structure characterization could demonstrate, in advance, the photocatalytic activity of BiVO_4_/Mn_1−*x*_Zn_*x*_Fe_2_O_4_/RGO to some extent. 

### 3.3. Magnetic Performance and Optical Properties

The magnetic hysteresis loops of the samples were displayed in Figure 6. The saturation magnetization (Ms) of Mn_1−*x*_Zn_*x*_Fe_2_O_4_ and BiVO_4_/Mn_1−*x*_Zn_*x*_Fe_2_O_4_/RGO were 84.03 and 8.21 emu g^−1^, respectively. Ms of the compounds was lower than that of the pure Mn_1−*x*_Zn_*x*_Fe_2_O_4_, owing to the amount decrease of the magnetic substance quantity in per unit composite. It was obvious that the prepared composite BiVO_4_/Mn_1−*x*_Zn_*x*_Fe_2_O_4_/RGO had a soft-magnetic feature like pure Mn_1−*x*_Zn_*x*_Fe_2_O_4_, which further confirmed than the synthesized composite must be comprised of Mn_1−*x*_Zn_*x*_Fe_2_O_4_ component [20,26].

It was worth noting that Ms was no attenuation after BiVO_4_/Mn_1−*x*_Zn_*x*_Fe_2_O_4_/RGO was employed after five rounds of recycling, indicating the stable magnetism of the as-prepared composite photocatalyst. More importantly, the compound exhibited outstanding paramagnetism because both coercivity (Hc) and remnant magnetization (Mr) were near to zero. Obviously, the excellent magnetic property ensured the high recovery ratio of BiVO_4_/Mn_1−*x*_Zn_*x*_Fe_2_O_4_/RGO using an external magnet after reaction.

**Figure 7:**
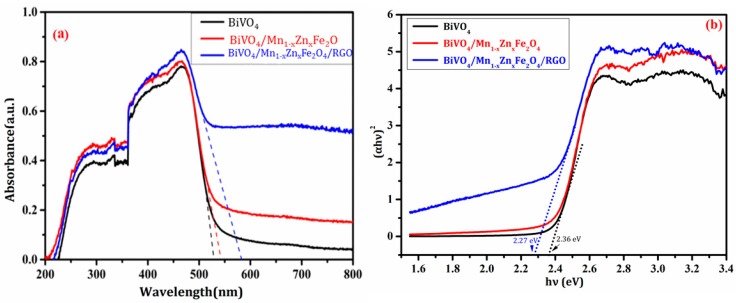
UV–vis diffuse reflectance spectra of the as-prepared products (**a**) and corresponding the plot of (Ahυ)^1/2^ versus hυ (**b**).

**Figure 8:**
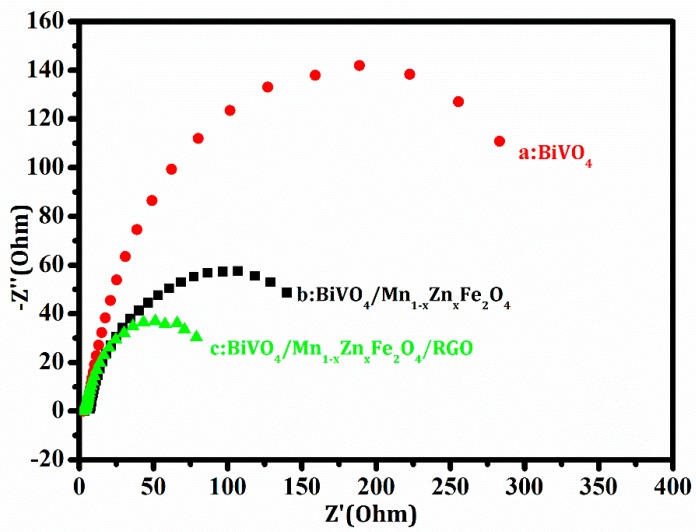
EIS of the work electrode containing BiVO_4_ (**a**); BiVO_4_/Mn_1−*x*_Zn_*x*_Fe_2_O_4_ (**b**) and BiVO_4_/Mn_1−*x*_Zn_*x*_Fe_2_O_4_/RGO (**c**).

The light absorption ability of the as-prepared samples was investigated with UV–vis DRS, and the diffuse reflectance spectra were recorded in Figure 7. It was seen from Figure 8a that the maximum absorption wavelength (λ_max_) of pure BiVO_4_ was about 500 nm. The further insights revealed the absorbance (at λ_max_ = 500 nm) of the compounds was higher than that of BiVO_4_. The band gap energy (E_g_) was estimated from (Ahv)^1/2^ ~ hv plots [5] (Figure 7b). E_g_ of BiVO_4_, BiVO_4_/Mn_1−*x*_Zn_*x*_Fe_2_O_4_, and BiVO_4_/Mn_1−*x*_Zn_*x*_Fe_2_O_4_/RGO were approximately 2.36 eV, 2.36 eV, and 2.27 eV, respectively. The introduction of Mn_1−*x*_Zn_*x*_Fe_2_O_4_ did not extend the absorbance light range of BiVO_4_ [20]. However, the introduction of RGO could be conducive to lessen E_g_, leading to the enhancement of visible light absorbance for BiVO_4_/Mn_1−*x*_Zn_*x*_Fe_2_O_4_. It is true that the great light absorption was closely related to good photocatalytic activity of catalysts [26].

### 3.4. Electrochemical Performance

Electrochemical impedance spectroscopy (EIS) was an effective approach to evaluate electron transfer ability in the interface between solid phase electrodes and electrolyte solution [28]. The typical impedance spectra of the samples were displayed with Nyquist plots. The semicircle diameter in Figure 8 became small when RGO inserted in the work electrode contained the compound. This change implied the resistance decrease and the conductivity increase in the test interface. The charge-transfer resistance (R_ct_) of the samples was gained by fitting the data from Figure 8. R_ct_ of BiVO_4_, BiVO_4_/Mn_1−*x*_Zn_*x*_Fe_2_O_4_, and BiVO_4_/Mn_1−*x*_Zn_*x*_Fe_2_O_4_/RGO were 351.0 Ω cm^2^, 206.0 Ω cm^2^, and 103.0 Ω cm^2^, respectively. It was clear that R_ct_ of the ternary composite was the lowest.

The good electron accepting and transporting properties of RGO could contribute to the prevention of charge recombination. It was reasonable that the introduction of RGO was beneficial to the efficient charge separation and transportation in the compound interface. The electrochemical behavior brought high conductivity of the comprising electrode. As a result, the enhancement of conductivity promoted the improvement of photocatalytic activity for BiVO_4_/Mn_1−*x*_Zn_*x*_Fe_2_O_4_/RGO.

### 3.5. Photocatalytic Activity, Stability, and Corresponding Mechanism

**Figure 9:**
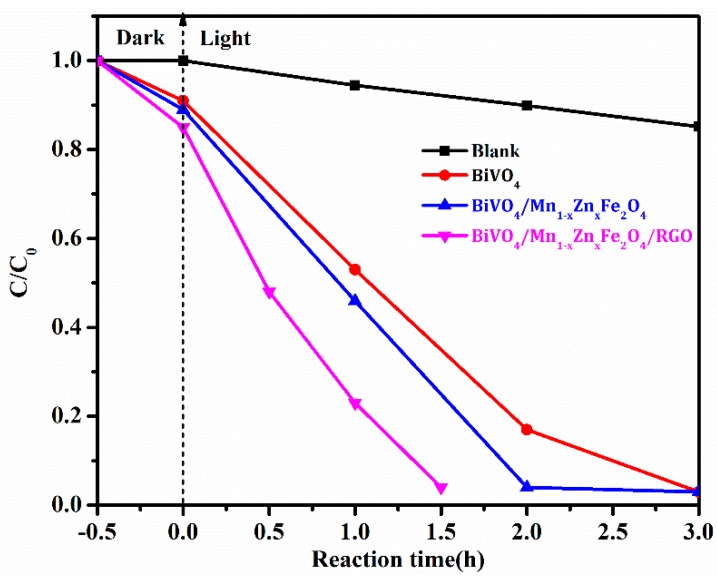
Degradation ratios of rhodamine B (RhB) with photocatalysts.

The photocatalytic activity was probed with photodegradation of RhB dye, and the result was shown in Figure 9. It was found from Figure 9 that the degradation ratio of RhB with BiVO_4_/Mn_1−*x*_Zn_*x*_Fe_2_O_4_/RGO under visible light irradiation reached to 96.0% after only 1.5 h reaction. It is worth noting that the self-degradation of RhB was very weak in the comparative test. It took about 3 h to get the same degradation ratio (96.0%) with pure BiVO_4_ as well as BiVO_4_/Mn_1−*x*_Zn_*x*_Fe_2_O_4_ under identical conditions. Significantly, BiVO_4_/Mn_1−*x*_Zn_*x*_Fe_2_O_4_/RGO exhibited more excellent photocatalytic activity than that of BiVO_4_/Mn_1−*x*_Zn_*x*_Fe_2_O_4_. Moreover, the activity of BiVO_4_/Mn_1−*x*_Zn_*x*_Fe_2_O_4_/RGO was greatly superior to that of SrFe_12_O_19_/BiVO_4_, as well as BiVO_4_/RGO, in the literature [19,22]. The high photocatalytic property of the as-produced compound BiVO_4_/Mn_1−*x*_Zn_*x*_Fe_2_O_4_/RGO was explained as follows: the graphene owned two-dimensional π–π conjugate structure was not only a good electron acceptor, but also a good electronic vector. RGO excited electrons in BiVO_4_/Mn_1−*x*_Zn_*x*_Fe_2_O_4_ and prompted the transferring of the conduction band in itself [29]. It was more interesting that the photocatalytic activity of BiVO_4_/Mn_1−*x*_Zn_*x*_Fe_2_O_4_/RGO was obviously better than that of Mn_1−*x*_Zn_*x*_Fe_2_O_4_/β-Bi_2_O_3_ in previous our group’s report [20].

**Figure 10:**
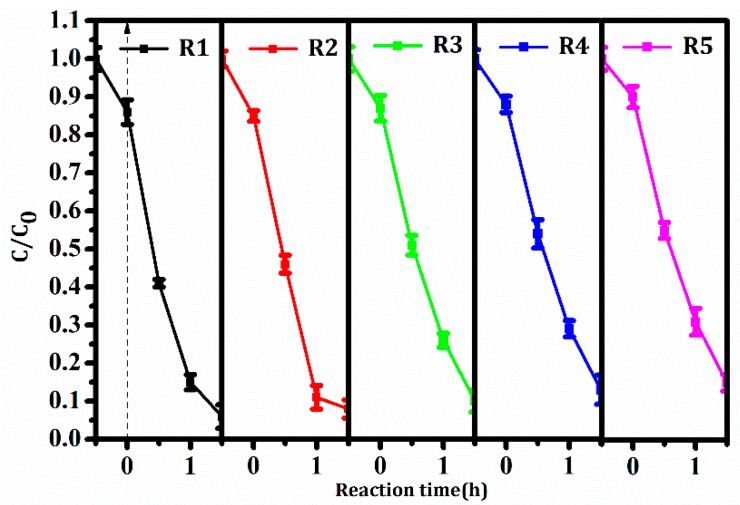
Cycling test of BiVO_4_/Mn_1−*x*_Zn_*x*_Fe_2_O_4_/RGO in RhB photodegradation (five recycles).

The stability was a key property in the industrial application of catalytic materials. Each recycling experiment was operated in triplicate, and average values and standard deviations were also shown in Figure 10. The degradation ratio of RhB in the fifth recycling was still 85.0% after 1.5 h of reaction under the same test parameters. The photocatalytic activity was only reduced a little within five recycles. The result revealed good stability of BiVO_4_/Mn_1−*x*_Zn_*x*_Fe_2_O_4_/RGO.

**Figure 11:**
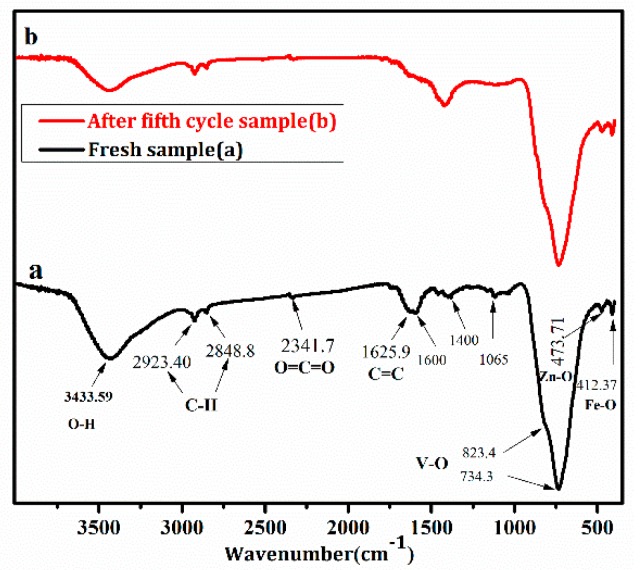
FTIR spectra of Mn_1−*x*_Zn_*x*_Fe_2_O_4_/BiVO_4_/RGO (**a**) the fresh sample; (**b**) the recovered sample.

Figure 11 was FTIR spectra of the compounds. The peaks at 473.7 cm^−1^ and 412.4 cm^−1^ in Figure 11a,b were assigned to Zn–O and Fe–O vibrations in Mn_1−*x*_Zn_*x*_Fe_2_O_4_. Characteristic patterns of V–O symmetric and asymmetric stretching vibrations spectra in BiVO_4_ were present at 734.3 cm^−1^ and 823.4 cm^−1^. The abovementioned peak location and intensity of Mn_1−*x*_Zn_*x*_Fe_2_O_4_ and BiVO_4_ were not varied, demonstrating a high stability of BiVO_4_/Mn_1−*x*_Zn_*x*_Fe_2_O_4_ during the photocatalytic reaction. The peak at 1625.9 cm^−1^ in Figure 11 belonged to C=C stretch of aromatic group in RGO. The peak at 1629.5 cm^−1^ in Figure 11b was weaker than that in Figure 11a, due to a little loss of RGO quantity after the fifth cycle. The absorption peaks located at around 1400.0 cm^−1^ and 1065.0 cm^−1^ illustrated the functional group of RGO [30]. By comparing pattern (a) and (b) in Figure 11, the typical peaks were detected in the spectra of the fresh, as well as the recovered compound. Thereby, it was concluded that the structure of BiVO_4_/Mn_1−*x*_Zn_*x*_Fe_2_O_4_/RGO was stable in the process of RhB photocatalytic degradation.

**Figure 12:**
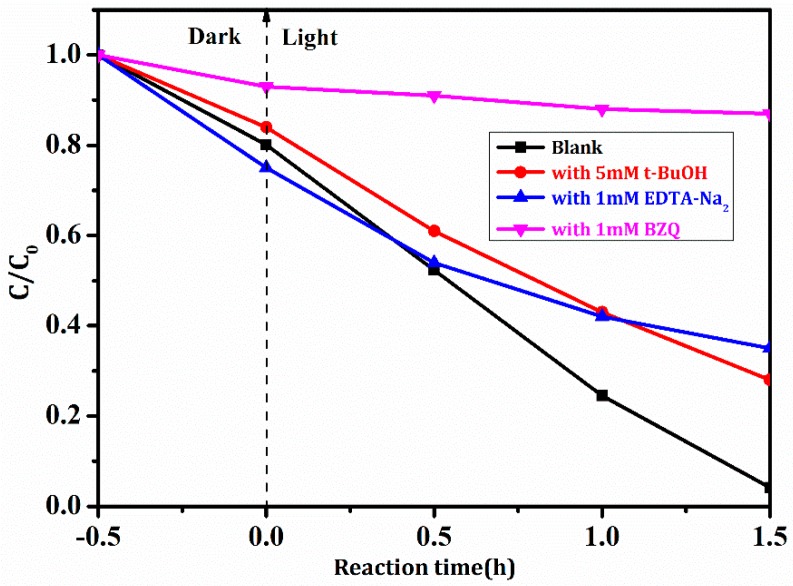
Photodegradation ratios of RhB with BiVO_4_/Mn_1−*x*_Zn_*x*_Fe_2_O_4_/RGO under scavengers.

The photocatalytic mechanism of BiVO_4_/Mn_1−*x*_Zn_*x*_Fe_2_O_4_/RGO was probed with radical scavengers [31]. *t*-BuOH (•OH scavenger), EDTA-Na_2_ (h^+^ scavenger), and BZQ (•O_2_^−^ scavenger) were employed to ascertain the dominant radical species in the photocatalytic degradation of RhB with the as-synthesized compound. Degradation ratios of RhB under these scavengers were given in Figure 12. The photodegradation ratio of RhB was 72.0% and 65.0% only when 5.0 mM *t*-BuOH and 1.0 mM EDTA-Na_2_ were added into the reaction system. Namely, h^+^ or •OH scavenger brought about the decrease of degradation ratios. Thus, the photocatalytic activity of the compound greatly decreased. The inactivation of the photocatalytic test was evidently proven when 1 mM BZQ was added in the same reaction system. The above results demonstrated that the most active species was •O_2_^−^, though •OH and h^+^ also contributed to the photocatalytic activity of BiVO_4_/Mn_1−*x*_Zn_*x*_Fe_2_O_4_/RGO.

**Figure 13:**
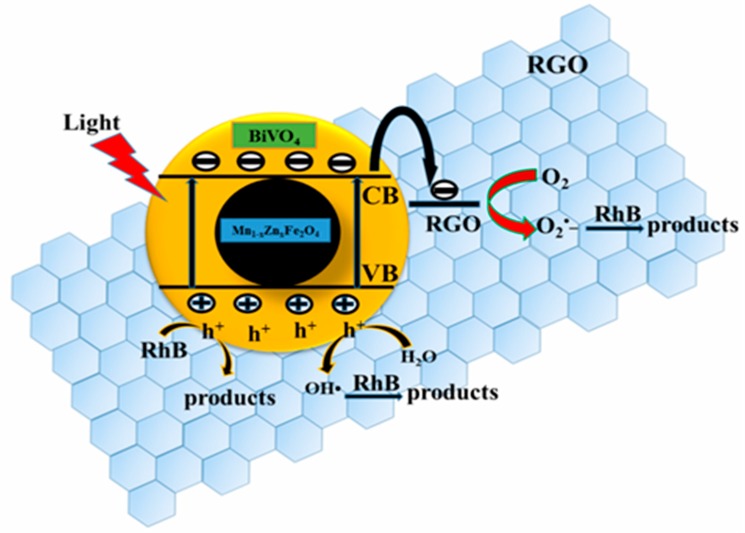
Photocatalytic scheme of BiVO_4_/Mn_1−*x*_Zn_*x*_Fe_2_O_4_/RGO under visible light irradiation.

Figure 13 described the electron–hole pairs forming process under the light irradiation. The potentials of conduction band (CB) and valence band (VB) of BiVO_4_ were 0.46 eV and 2.86 eV, respectively (referring to hydrogen electrode, NHE). The electrons in VB were excited to CB under visible light irradiation, forming driven-electrons (e^−^) and holes (h^+^). The VB potential of BiVO_4_ was close to E^θ^ of •OH/H_2_O, and the CB potential was larger than E^θ^ of O_2_/•O_2_^−^. This meant that electrons were able to directly reduce O_2_ molecules into superoxide O_2_^−^. Besides, RhB molecules were directly oxidized by the holes on VB of BiVO_4_. In addition, there were much more active adsorption centers and photocatalytic reaction sites in RGO with a large surface area. These active centers and sites were beneficial to the improvement of the photocatalytic activity. As a good electron accepter and electronic vector, RGO facilitates the transmission of photo-produced electrons, which was conducive to the separation of photo-produced electrons and holes, and further promoted the formation of •O_2_^−^. It was ensured that •O_2_^−^ played the main role for the RhB photodegradation, though •OH and h^+^ had a collaborative oxidation role in the photocatalytic reaction of BiVO_4_/Mn_1−*x*_Zn_*x*_Fe_2_O_4_/RGO.

In fact, since our group found that the magnetic composite ZnFe_2_O_4_/SrFe_12_O_19_ had a highly photocatalytic activity in 2013 [27], we firmly thought that the stable magnetic field from SrFe_12_O_19_ itself could promote the separation of photo-generated electrons and holes, and furthermore, that the photocatalyst could produce more photo-generated electrons and holes under identical light irradiation. Thus, the photoelectric transformation efficiency would be boosted. Our group the studied BiVO_4_/SrFe_12_O_19_, Bi_2_O_3_/SrFe_12_O_19_, and BiOCl/SrFe_12_O_19_ [22,32,33] magnetic heterojunction to confirm previous speculation. However, these studies about its photoelectron transfer mechanism were not enough. In future work, our group will continue to attempt to confirm our speculation via experimental and theoretical calculations. Of course, this work was carried out in order to compare with SrFe_12_O_19_ functions.

## 4. Conclusions

Magnetic photocatalyst BiVO_4_/Mn_1−*x*_Zn_*x*_Fe_2_O_4_/RGO was synthesized with the simple and economical roasting-reduction approach. The photocatalyst exhibited excellent photocatalytic activity and stability. The degradation ratio of RhB reached 96.0% under visible light irradiation after only 1.5 h reaction with the photocatalyst. The degradation ratio of RhB was still maintained at 85.0% after five cycles of photocatalytic reaction. Here, the incorporation of RGO enhanced the specific surface area compared with BiVO4/Mn_1−*x*_Zn_*x*_Fe_2_O_4_.The larger specific surface area provided more active surface sites, more free space to improve the mobility of photo-induced electrons, and further facilitated the effective migration of charge carriers, leading to the remarkable improvement of photocatalytic performance. RGO was the effective acceptor as well as transporter of photo-generated electron–hole pairs. •O_2_^−^ was the most active species in this photocatalytic reaction. We hope this photocatalyst has a wide application in organic contaminants removal or environmental pollution control in practical.
